# Anti-cytokine autoantibodies in postherpetic neuralgia

**DOI:** 10.1186/s12967-015-0695-6

**Published:** 2015-10-20

**Authors:** Ahmad Bayat, Peter D. Burbelo, Sarah K. Browne, Mark Quinlivan, Bianca Martinez, Steven M. Holland, Asokumar Buvanendran, Jeffrey S. Kroin, Andrew J. Mannes, Judith Breuer, Jeffrey I. Cohen, Michael J. Iadarola

**Affiliations:** Department of Perioperative Medicine, Clinical Center, National Institutes of Health, Bethesda, MD 20892 USA; Dental Clinical Research Core, National Institute of Dental and Craniofacial Research, National Institutes of Health, Bethesda, MD 20892 USA; Laboratory of Clinical Infectious Diseases, National Institute of Allergy and Infectious Diseases, National Institutes of Health, Bethesda, MD 20892 USA; Division of Infection and Immunity, University College London, London, WC1E 6BT UK; Department of Anesthesiology, Rush University Medical Center, Chicago, IL 60612 USA; Laboratory of Infectious Diseases, National Institute of Allergy and Infectious Diseases, National Institutes of Health, Bethesda, MD 20892 USA

**Keywords:** Anti-cytokine autoantibodies, Herpes zoster (HZ), Opportunistic infection, Pain, PHN, Shingles, Varicella zoster virus (VZV)

## Abstract

**Background:**

The mechanisms by which varicella zoster virus (VZV) reactivation causes postherpetic neuralgia (PHN), a debilitating chronic pain condition, have not been fully elucidated. Based on previous studies identifying a causative role for anti-cytokine autoantibodies in patients with opportunistic infections, we explored this possibility in PHN.

**Methods:**

Sera from herpes zoster (HZ) patients without and with PHN (N = 115 and 83, respectively) were examined for the presence of autoantibodies against multiple cytokines, and other known autoantigens. In addition, a cohort of patients with complex regional pain syndrome or neuropathic pain was tested for autoantibodies against selected cytokines. Antibody levels against VZV, Epstein Barr virus, and herpes simplex virus-2 were also measured in the HZ and PHN patients. Patient sera with high levels of anti-cytokine autoantibodies were functionally tested for in vitro neutralizing activity.

**Results:**

Six PHN subjects demonstrated markedly elevated levels of single, autoantibodies against interferon-α, interferon-γ, GM-CSF, or interleukin-6. In contrast, the HZ and the pain control group showed low or no autoantibodies, respectively, against these four cytokines. Further analysis revealed that one PHN patient with high levels of anti-interleukin-6 autoantibodies had a markedly depressed antibody level to VZV, potentially reflecting poor T cell immunity against VZV. *In vitro* functional testing revealed that three of the five anti-cytokine autoantibody positive PHN subjects had neutralizing autoantibodies against interferon-α, GM-CSF or interleukin-6. In contrast, none of the HZ patients without PHN had neutralizing autoantibodies.

**Conclusions:**

These results suggest the possibility that sporadic anti-cytokine autoantibodies in some subjects may cause an autoimmune immunodeficiency syndrome leading to uncontrolled VZV reactivation, nerve damage and subsequent PHN.

## Background

Varicella-zoster virus (VZV) is a member of the alpha-herpes virus subfamily that causes chickenpox early in life and then remains latent in neurons of sensory ganglia for the lifetime of the individual [1]. In persons with immune deficiencies and in older individuals with a waning immune system, VZV can reactivate causing herpes zoster (HZ), also known as shingles [2]. HZ patients have vesicular, unilateral, painful, dermatomally demarcated skin eruptions that typically resolve in less than 1 month. HZ is quite frequent, and approximately one third of the population may experience zoster once in their lifetime [3]. Adults over the age of 50 are particularly susceptible to HZ [1], implicating a stronger role of acquired factors compared to genetic influences [4]. One potential consequence of HZ is the development of postherpetic neuralgia (PHN), defined as pain that lasts for greater than 3 months and which originates in the same area as the HZ rash. The frequency of developing PHN has been reported to be approximately 10 % after zoster [5, 6]. Immunocompromised individuals are at higher risk of HZ [7, 8], suggesting an important role of immune surveillance in controlling VZV infection. The Shingles Prevention Study showed that vaccination of elderly persons with a live attenuated varicella-zoster virus vaccine reduced the rates of both zoster and PHN [9]. In a follow-up study, Weinberg et al. showed higher cell-mediated immunity at HZ onset, which correlated with reduced HZ severity and less PHN; in contrast humoral immunity, as reflected by antibodies to VZV at onset of HZ did not correlate with severity of disease [10]. These results indicate a critical function of T-cell-mediated immunity for protection against HZ and PHN.

Recently, we explored the role of anti-cytokine autoantibodies in several diseases by screening patient sera with a panel of recombinant *Renilla* luciferase cytokine fusion proteins as antigenic probes using the Luciferase Immunoprecipitation Systems (LIPS) technology [11–13]. Using this approach, thymoma patients with opportunistic infections, including some with disseminated VZV infection, demonstrated autoantibodies against interferon-α (IFN-α), interleukin 12p35 (IL-12p35), and several other cytokines [12]. High levels of neutralizing anti-IFN-γ autoantibodies were also detected in patients with disseminated nontuberculous mycobacteria and other opportunistic infections, including both with localized and disseminated VZV reactivation [14, 15]. Based on the late age of onset of PHN, we explored in this study whether anti-cytokine and other autoantibodies, might be associated with PHN. From screening a cohort of HZ patients with and without PHN, high levels of autoantibodies against several different cytokines were detected in six PHN patients. Further analysis revealed that three of the PHN patients had neutralizing anti-cytokine autoantibodies. In one PHN patient with high level anti-IL-6 autoantibodies, antibody responses against VZV were completely absent. The finding that several patients each harbored single, neutralizing autoantibodies against interferon-α, GM-CSF or IL-6 suggests that anti-cytokine immunodeficiency may contribute to development of PHN.

## Methods

### Subjects

Informed written consent was obtained from all subjects with VZV reactivation in accordance with the Human Experimentation Guidelines of University College London, (East London and the city Research Ethics Committee LREC R&WF2002/38). A total of 198 HZ subjects were studied: 115 without PHN (hereafter referred to as HZ) and 83 with PHN (hereafter referred to as PHN). There were 28 subjects with HZ who had a dysesthesia, but no PHN; using our measured endpoints there were no differences between patients with HZ and those with HZ and dysesthesia and these two groups were analyzed together. Two additional subject groups were studied as controls. First, a small group of healthy blood donor controls (n = 8) were used to standardize the assay. Second, a PHN age-matched disease control group (n = 50), from patients having either complex regional pain syndrome (CRPS) or neuropathic pain (NP) were tested for selected anti-cytokine autoantibodies. The CRPS/NP subjects were collected at Rush University under an IRB approved protocol and with patient written consent. The clinical characteristics of these four different groups of subjects including the age, gender, diagnosis, and the presence of other associated immunodeficiencies, are described in Table 1.

**Table 1:** Demographic information for the different study groups

	Number	Age (mean ± SD)	Gender (M/F)	Immune status		
				Competent	Compromised	On inhaled steroids
HZ^a^	115	47 ± 18.8	54/61	94	17	4
PHN	83	61 ± 16.9	55/28	49	27	7
Healthy controls	8	39 ± 18.0	3/5	8	0	0
CRPS/NP	50	60 ± 8.1	27/23	50		

^a^Includes 28 HZ patients with dysesthesia, but no PHN

### LIPS antigens and screening

The antigen targets used for LIPS testing have been previously described [12–14]. An initial autoantibody screen of twenty-four potential autoantigens was performed with a pilot set of 33 PHN patients and 8 healthy controls. These included 13 cytokines (GM-CSF, IL-6, IFN-α, IL-12-p35, IFN-γ, IFN-ω, IFN- λ, IL-12-p40, IL-10, TNF-α, TNF-β, IL-17 and IL-1α), six known autoantigens (Ro52, Ro60, La, RNP-A, Sm-D3 and RNP-70), four neuro-glial proteins, glutamic acid decarboxylase (GAD-65), tyrosine hydroxylase, S100-β, and aquaporin-4, and TRIM-15. Based on the results of the pilot study, 168 additional HZ patients with and without PHN were tested against the most informative targets (Ro52, Ro60, IFN-α, IFN-γ, IL-6, and GM-CSF). All the subjects in the cohort also were evaluated for antibodies against BLRF2 of EBV (p23 capsid), the gE glycoprotein of VZV [16] and the gG-2 glycoprotein of HSV-2 [17].

For LIPS autoantibody testing, serum samples were diluted 1:10 in assay buffer A (20 mM Tris, pH 7.5, 150 mM NaCl, 5 mM MgCl_2_, 1 % Triton X-100), arrayed in 96 deep well microtiter plates, and tested as described [17]. Buffer blanks were used to monitor the performance and background binding activity of the assays. Light units (LU) were measured using a Berthold luminometer and all LU data were obtained from the average of at least two separate experiments.

### Detection of neutralization activity for anti-cytokine autoantibodies

To evaluate the neutralizing capacity of anti-cytokine autoantibodies, control PBMCs from individual, healthy blood donors, that were distinct from the 8 controls used for antibody analysis, were incubated in the presence of 10 % healthy control or patient sera and left unstimulated or stimulated with the cytokine recognized by the autoantibodies in that particular patient sample. The PBMC were fixed, permeabilized and stained for an increase in phosphorylation of the specific downstream Signal Transducer and Activator of Transcription (pSTAT) molecules by flow cytometry as described previously [14]. Thus, for patients with anti-IFN-α autoantibodies, cells were stimulated with IFN-α (1000 U/mL) and assessed for IFN-α-induced pSTAT-1 in CD14 + monocytes; those with anti-IFNγ-autoantibodies were stimulated with IFN-γ (1000 U/mL) induced pSTAT-1 in CD14 + monocytes; those with anti-GM-CSF autoantibodies for GM-CSF (10 ng/mL)-induced pSTAT-5 in CD14+ monocytes; those with anti-IL-6 autoantibodies for IL-6 (10 ng/mL) induced pSTAT-3 in CD3+ lymphocytes. Antibodies for flow cytometry were purchased from BD Biosciences. Data were collected using FACSCanto (BD Biosciences) and analyzed using FlowJo Version 9.1 (TreeStar). Using this approach as described [12, 14, 18, 19], the magnitude of pSTAT production due to cytokine stimulation is extremely reproducible and sensitive to incremental changes in the amount of cytokine added and sera was categorized as not neutralizing, partially neutralizing and neutralizing.

### Statistical analysis

GraphPad Prism software (San Diego, CA, USA) was used for analyzing the antibody levels in this study. Geometric mean antibody levels, expressed as mean log (10) LU and 95 % confidence intervals (CI), were calculated and presented as antilog values. The non-parametric Mann–Whitney *U* statistical test was used for comparison of antibody levels in the control and patient groups. The Fischer exact test was used to evaluate the statistical significance of the prevalence of anti-cytokine autoantibodies in the HZ and PHN subjects.

## Results

### Anti-cytokine autoantibodies in subjects with HZ and PHN

An initial autoantibody screen of twenty-four potential autoantigens was performed with a pilot set of 33 PHN patients and 8 healthy controls. Seropositivity was observed as follows: 15 % (5/33) for Ro52 and La and 12 % (4/33) for anti Ro60 autoantibodies. Antibody levels for these three autoantigens were often 1000-fold higher than the buffer blanks or other seronegative samples. We also observed that four PHN patients showed unusually high levels of autoantibodies against single cytokines including IFN-γ, GM-CSF and IL-6. Additional weak positive autoantibodies were detected against IFN-ω and AQP-4 in several healthy controls and PHN samples. Autoantibodies against RNP-70, Sm-D3, IFN-λ, TNF-α, TNF-β, IL-17, IL-10, IL-12-p35, IL-12-p40, S100β and GAD-65 were not found in the pilot cohort.

**Fig. 1:**
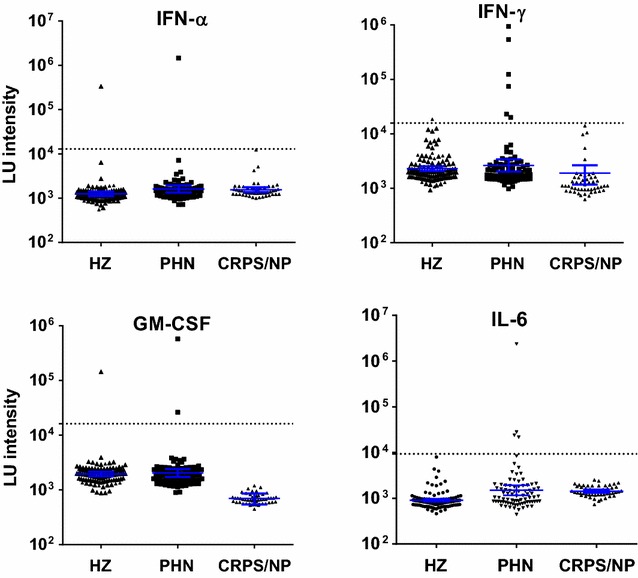
Anti-cytokine autoantibodies in herpes zoster (HZ), post-herpetic neuralgia (PHN) and complex regional pain/neuropathic pain (CRPS/NP) subjects. Each point represents an individual sample from the cohorts composed of 248 samples divided into three groups: HZ (N = 115), PHN (N = 83), and CRPS/NP (N = 50). All samples were screened for autoantibodies to IFN-α, IFN-γ, IL-6 and GM-CSF by LIPS. The *blue lines* represent the geometric mean with 95 % CI. The *dotted line* is the cutoff value

Based on these findings, an additional 115 subjects with only HZ and 50 PHN subjects were evaluated with the most informative autoantigens (IFN-α, IFN-γ, GMCSF, IL-6, Ro52 and Ro60) identified in the pilot cohort. In this larger group of samples, ten more of the PHN patients were seropositive for anti-cytokine autoantibodies. For analysis and presentation, both the pilot and second set of samples were merged. As shown in Fig. 1a, only three HZ patients demonstrated single, seropositive anti-cytokine autoantibodies that were directed against IFN-α, IFN-γ, and GM-CSF. In total, the PHN subjects contained thirteen seropositive anti-cytokine autoantibodies, which as a group was statistically different than the HZ (Fischer’s Exact test *P* = 0.001). Moreover, an age-matched disease control cohort of CRPS/NP patients (n = 50) failed to harbor any statistically significant autoantibody responses against IFN-α, IFN-γ, GM-CSF or IL-6 (Fig. 1). Particularly relevant was the finding of six PHN patients (one against anti-IFN-α, three against IFN-γ, one against GM-CSF, and one against IL-6) with high levels of autoantibodies greater than 100,000 LU that might have potential neutralizing activity against the target cytokine (Fig. 1). Besides cytokine autoantibodies, five PHN patients had robust autoantibodies against Ro52 and four PHN patients with Ro60 autoantibodies, in which three of the PHN patients were co-positive for both Ro52 and Ro60 (data not shown). Inspection of the individual patients revealed that only one of the low IL-6 autoantibody seropositive PHN subjects was copositive for Ro52 or Ro60, suggesting that general polyreactivity did not underlie the immunoreactivity seen in anti-cytokine autoantibody patients. Lastly, review of the patients’ charts in subjects with anti-cytokine autoantibodies did not document the presence of thymoma, *Staphylococcus* infection, or any other unusual condition to explain these autoantibodies. None of the patients had other known autoimmune diseases associated with anti-cytokine autoantibodies including autoimmune polyendocrinopathy candidiasis ectodermal dystrophy (APECED) or pulmonary alveolar proteinosis (PAP).

### Levels of antibodies against VZV and other herpesviruses in the cohort

**Fig. 2:**
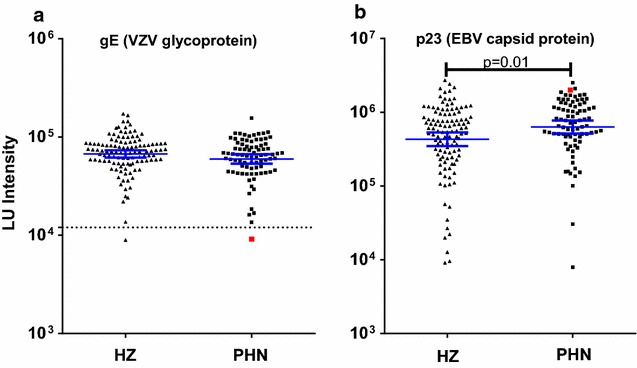
Evaluation of antibodies against VZV and EBV in the HZ and PHN cohorts. Serum samples were assayed for antibodies against VZV and EBV by LIPS. **a** The antibody levels against the gE glycoprotein antigen of VZV and **b** antibody levels to the p23 capsid protein of EBV in the HZ and PHN groups. For VZV antibodies, the *dotted line* represents the previously established cutoff value [16]. The *red square* corresponds to patient #226 (see Table 2) who is the IL-6 seropositive PHN patient who was seronegative for VZV antibodies

In addition to studying autoantibodies, we evaluated the possibility that the levels of VZV antibodies might be altered between HZ and PHN. For serologic testing, we measured antibodies against gE, a known viral glycoprotein target of natural and vaccine-mediated immunity [16]. As shown in Fig. 2a, there were no statistical differences in the gE antibody levels in subjects with HZ compared to PHN. Several HZ and PHN subjects had very low or no anti-VZV antibodies (Fig. 2a). However, further inspection of the individual patients with anti-cytokine autoantibodies revealed that one PHN patient, #226, with high levels of anti-IL-6 autoantibodies was seronegative, having the lowest level of antibodies against VZV in the PHN patients (Fig. 2a). The two other subjects with neutralizing autoantibodies against IFN-α and GM-CSF had VZV antibodies in the normal range **(**Fig. 2a**)**. To explore whether these blunted antibody responses were unique only to VZV, antibodies against another herpes virus, EBV, using the p23 capsid protein were measured. The average antibody level to the p23 capsid of EBV was significantly higher (P = 0.01) in the PHN group compared to the HZ group **(**Fig. 2b). Antibodies to a third herpes virus, HSV-2, were too sporadic to be informative (only 13 % of the entire cohort (26/198 patients) exhibited HSV-2 seropositivity (data not shown). Taken together, viral antibody profiling suggests that, as a group, these patients are not globally immunocompromised yet at a more fine-grained level, patient #226, who showed severely blunted VZV antibodies, had high levels of anti-EBV antibodies.

### Detection of neutralizing anti-cytokine autoantibodies in a few PHN patients

Based on our previous studies [12, 14], the eight patients in Fig. 1a with high levels of anti-cytokine autoantibodies (>100,000 LU) were selected to test for their in vitro inhibitory capacities to block the activity of the cognate cytokine (Table 2). Serum from HZ patient #6 partially inhibited IFNα-induced pSTAT-1, and serum from PHN patient #56 completely prevented IFN-α-induced pSTAT-1 (Table 2). For the three patients with high levels of IFN-γ autoantibodies, PHN patient #123 and #322 did not block IFNγ-induced pSTAT-1 whereas serum from the third PHN patient, #207, was partially blocking (Table 2). PHN patient #205 with anti-GM-CSF autoantibodies prevented GM-CSF-induced pSTAT-5 production, while serum from HZ patient #215 with lower levels of anti-GM-CSF autoantibodies did not. Testing the PHN patient #226 with the sole anti-IL-6 autoantibodies revealed that serum from this individual prevented IL-6-induced pSTAT-3. In total, only three PHN patients had serum autoantibodies that had complete neutralizing activity against the cognate cytokine.

**Table 2:** Neutralizing activity of anti-cytokine autoantibodies in HZ and PHN

Cytokine	Pt. ID	Group	Ab level (LU)^a^	Other samples^a^ (95 % CI)	Neutralizing activity^b^
IFN-α	56	PHN	1,465,000	1388 ± 89 (LU)	*Neutralizing*
IFN-α	6	HZ	335,000	1388 ± 89 (LU)	Partially neutralizing
IFN-γ	123	PHN	125,000	2943 ± 535 (LU)	Not neutralizing
IFN-γ	207	PHN	541,000	2943 ± 535 (LU)	Partially neutralizing
IFN-γ	322	PHN	939,000	2943 ± 535 (LU)	Not neutralizing
IL-6	226	PHN	2,371,000	1555 ± 419 (LU)	*Neutralizing*
GM-CSF	205	PHN	575,000	2079 ± 252 (LU)	*Neutralizing*
GM-CSF	215	HZ	145,000	2079 ± 252 (LU)	Not neutralizing

^a^Antibody levels determined by LIPS

^b^Cytokine neutralization activity as described in the methods

## Discussion

Chronic pain conditions are frequently life-long problems that are difficult to treat, in part, because the underlying factors that contribute to human pain disorders are unclear. For PHN, the mechanisms that contribute to the transition from zoster to PHN remain nebulous. While animal models of PHN have been explored they have many limitations in reproducing the multiple factors encountered in the human disease [20]. Thus, clinical research efforts based directly in human subjects are needed to obtain mechanistic insights into PHN. Based on our earlier work [12, 14, 18, 19], the potential contributions of humoral immune processes to HZ and PHN were investigated with a focus on autoantibodies against cytokines and several known neural and non-neural autoantigens. We further hypothesized that damage to the central or peripheral nervous systems may expose autoantigenic epitopes thereby provoking an autoimmune response [21]. Several reports have appeared examining the presence of autoantigens to peripheral nerve in complex regional pain syndrome [22, 23] and to central nervous system proteins in disorders such multiple sclerosis, although results for some antigens are controversial [24]. In PHN, significant shrinkage in the dorsal horn of the spinal cord has been observed that might be attributable to injury and potential autoimmune attack [25]. Using LIPS, no autoantibodies were detected against two neural antigens, GAD-65 and tyrosine hydroxylase, or two astrocyte antigens S100-β and aquaporin-4 in any of the groups examined. While autoantibodies to four of the six Sjögren’s/systemic lupus erythematosus antigens tested (RNP-A, RNP-70, Sm-D3, and La), were also seronegative, a few patients exhibited elevated autoantibodies against Ro52 and Ro60. These data suggest that PHN, while a painful, stressful sequelae to an infectious disease process, does not induce an autoimmune state to the proteins tested. Previously, the induction of autoantibodies in acute respiratory distress syndrome (ARDS) and septic shock, conditions characterized by severe inflammation and cytokine storm were observed [26]. We hypothesized that the autoantibody induction was due to the ongoing systemic inflammation and associated tissue destruction that may mediate a break in tolerance against these self-proteins. In the ARDS study the levels of autoantibodies were substantially lower than those seen in the anti-cytokine positive patients identified here. This quantitative difference implies that in the PHN patients these anti-cytokine autoantibodies may be pathogenic rather than biomarkers for inflammation. Moreover, the antiviral humoral response against VZV and other HSVs was similar between the HZ and PHN patients. With the LIPS fluid-phase immunoassay used, our data indicate that the humoral response to VZV is intact in most subjects with HZ and PHN.

The novel finding of our study was the presence of sporadic anti-cytokine autoantibodies that were associated with PHN but not with HZ *per se*. We found six patients with PHN who had high levels (>100,000 LU) of anti-cytokine autoantibodies, but only two patients with HZ and none with CRPS/NP. In neutralization assays, only three subjects had serum antibodies that blocked cytokine signaling, all of whom had PHN. Two additional subjects, one with HZ and one with PHN, had partially neutralizing activities. The anti-IFN-α and anti-GM-CSF autoantibodies observed in two PHN patients may be associated with inflammation, as previously observed in ARDS and septic shock [26]. If the neutralizing levels of autoantibodies against these cytokines were present before or at the time of initial reactivation, then one might expect that they predispose to more severe VZV infection. Consistent with this hypothesis, patients with thymoma have both neutralizing anti-cytokine autoantibodies against IFN-α and a predisposition to both localized and disseminated varicella reactivation, raising the possibility that anti-cytokine autoantibodies might contribute to VZV reactivation in some cases [12]. The potential role of anti-GMCSF autoantibodies in generation of PHN is intriguing. Up until recently, anti-GMCSF autoantibodies were only thought to cause pulmonary alveolar proteinosis [27]. However, in the last several years, anti-GMCSF autoantibodies have been found in patients with cryptococcal meningitis [18, 28] and CNS infections with *Nocardia* bacteria [19]. In light of the finding of two subjects with VZV reactivation having elevated GMCSF autoantibodies, further investigations of the relationships between GMCSF and VZV infection are warranted.

The one PHN patient with very high levels of anti-IL-6 autoantibodies (i.e., 2,300,000 light units) that were neutralizing is potentially instructive. The presence of IL-6 autoantibodies in the general population, based on sampling of over 300 samples [12, 14] is quite rare. Consistent with a potential role for anti-IL-6 autoantibodies in promoting VZV reactivation, this PHN patient lacked antibodies against VZV, but showed normal humoral responses to EBV. It is possible that impaired IL-6 signaling blunts the inflammatory T-cell reaction and impairs adequate signaling to the relevant plasma cells for anti-VZV antibody production. High levels of anti-IL-6 autoantibodies were also noted in a child who developed an acute VZV infection complicated by multiple cellulitis lesions and subcutaneous *Staphylococcus aureus* abscesses [29]. Together these two patients implicate IL-6 autoantibodies in the modulation of primary and reactivation VZV infection. IL-6 is secreted by T cells and macrophages to stimulate the immune response and is known to be increased in the serum of HZ [30]. Mechanistically, VZV signals through Toll-like receptor-2-dependent activation of NF-kappa B to induce an efficient inflammatory response [31]. Low levels of IL-6 may lead to impaired anti-VZV immune responses, causing ineffective containment of VZV, more tissue damage and a stronger “peripheral generator” that then drives stronger central sensitization, all of which can contribute to PHN pain. Additional studies in more subjects with PHN are warranted to examine the possibility that IL-6 and other cytokine autoantibodies contribute to PHN.

There are several clinical implications of our findings. First, in sporadic cases, anti-cytokine autoantibodies may potentially contribute to PHN, highlighting the complex interactions between humoral immunity, T-cell immunity and cytokines. Based on the time line for the development of PHN from initial VZV reactivation, screening for anti-cytokine autoantibodies is theoretically possible, yet it is not clear how this might impact the course of treatment. The finding that the IL-6 cytokine might be involved in controlling VZV reactivation and thereby might be useful for early stage treatment of immunocompromised individuals warrants further investigation.

## Conclusions

It is likely that the development of PHN is a highly complex, multifactorial process driven by many subtle genetic, environmental and acquired factors. Based on the present observations, one such acquired factor is the presence of anti-cytokine autoantibodies, which could act as potential drivers of PHN by creating deficits in immune signaling. While the frequency of PHN patients harboring anti-cytokine autoantibodies found in our study was small, it is important to point out that PHN is quite common in adults over 60 and these findings would translate into explaining potentially many cases per year. Since autoantibodies and decline in immune system function are known to occur more commonly in older subjects [32–34], the present observations suggest the likelihood that there may be additional, unrecognized pathogenic autoantibodies against other immune components in older subjects that may increase susceptibility to PHN.
